# Toll-Like Receptor 21 of Chicken and Duck Recognize a Broad Array of Immunostimulatory CpG-oligodeoxynucleotide Sequences

**DOI:** 10.3390/vaccines8040639

**Published:** 2020-11-02

**Authors:** Yu-Chen Chuang, Jen-Chih Tseng, Jing-Xing Yang, Yi-Ling Liu, Da-Wei Yeh, Chao-Yang Lai, Guann-Yi Yu, Li-Chung Hsu, Chun-Ming Huang, Tsung-Hsien Chuang

**Affiliations:** 1 Immunology Research Center, National Health Research Institutes, Zhunan, Miaoli 35053, Taiwan; faseno@gmail.com (Y.-C.C.); mark0918@nhri.edu.tw (J.-C.T.); JingxingYang@nhri.edu.tw (J.-X.Y.); lil5410@nhri.org.tw (Y.-L.L.); daweiyeh@mail2000.com.tw (D.-W.Y.); 2 Department of Medical Laboratory Science and Biotechnology, Asia University, Taichung 41354, Taiwan; chaoyang@asia.edu.tw; 3 National Institute of Infectious Diseases and Vaccinology, National Health Research Institutes, Miaoli 35053, Taiwan; guannyiy@nhri.edu.tw; 4 Institute of Molecular Medicine, College of Medicine, National Taiwan University, Taipei 10002, Taiwan; lichunghsu@ntu.edu.tw; 5 Department of Biomedical Sciences and Engineering, National Central University, Taoyuan 32001, Taiwan; chunming@ncu.edu.tw; 6 Program in Environmental and Occupational Medicine, Kaohsiung Medical University, Kaohsiung 80708, Taiwan

**Keywords:** adjuvant, immune modulator, innate immunity, toll-like receptor, vaccine

## Abstract

CpG-oligodeoxynucleotides (CpG-ODNs) mimicking the function of microbial CpG-dideoxynucleotides containing DNA (CpG-DNA) are potent immune stimuli. The immunostimulatory activity and the species-specific activities of a CpG-ODN depend on its nucleotide sequence properties, including CpG-hexamer motif types, spacing between motifs, nucleotide sequence, and length. Toll-like receptor (TLR) 9 is the cellular receptor for CpG-ODNs in mammalian species, while TLR21 is the receptor in avian species. Mammalian cells lack TLR21, and avian cells lack TLR9; however, both TLRs are expressed in fish cells. While nucleotide sequence properties required for a CpG-ODN to strongly activate mammalian TLR9 and its species-specific activities to different mammalian TLR9s are better studied, CpG-ODN activation of TLR21 is not yet well investigated. Here we characterized chicken and duck TLR21s and investigated their activation by CpG-ODNs. Chicken and duck TLR21s contain 972 and 976 amino acid residues, respectively, and differ from TLR9s as they do not have an undefined region in their ectodomain. Cell-based TLR21 activation assays were established to investigate TLR21 activation by different CpG-ODNs. Unlike grouper TLR21, which was preferentially activated by CpG-ODN with a GTCGTT hexamer motif, chicken and duck TLR21s do not distinguish among different CpG-hexamer motifs. Additionally, these two poultry TLR21s were activated by CpG-ODNs with lengths ranging from 15 to 31 nucleotides and with different spacing between CpG-hexamer motifs. These suggested that compared to mammalian TLR9 and grouper TLR21, chicken and duck TLR21s have a broad CpG-ODN sequence recognition profile. Thus, they could also recognize a wide array of DNA-associated molecular patterns from microbes. Moreover, CpG-ODNs are being investigated as antimicrobial agents and as vaccine adjuvants for different species. This study revealed that there are more optimized CpG-ODNs that can be used in poultry farming as anti-infection agents compared to CpG-ODN choices available for other species.

## 1. Introduction

Chicken and duck are two major farmed avian species. Production loss caused by infectious diseases is a major problem in the poultry industry, thus, developing new strategies to combat infections is required for preventing massive losses [1,2,3]. Toll-like receptors (TLRs) are pattern-recognition receptors for detecting microbial pathogens. These are type I transmembrane receptors with an extracellular domain comprising multiple leucine-rich repeats, followed by a transmembrane region and a highly conserved cytoplasmic Toll/IL-1 receptor (TIR) domain. Ligand binding of these TLRs occurs within the ectodomain. The TIR domain provides a key site for homophilic interaction, with the TIR domain containing MyD88 family adapter proteins for activating NF-κB and IRF signaling pathways [4,5,6,7]. For avian TLRs, their downstream signaling molecules are relatively similar to those of mammalian TLRs, suggesting that avian TLRs might have a similar mechanism of action to their mammalian orthologs [8,9]. Activating these TLRs initiates early innate immunity and activates adaptive immunity for the host responses to invading microorganisms. Because of their potent immunostimulatory activity, different TLR agonists are being investigated as anti-infectious agents or as vaccine adjuvants for different species [10,11,12].

In mammals, 13 TLRs have been identified: humans possess 10 TLRs (TLR1–10), while mice have 13 TLRs (TLR1–13). In contrast, 20 TLRs have been identified in fishes [4,13,14,15]. Of the avian species, chicken (ch) TLRs are better studied, and 10 chTLRs (chTLR1La, chTLR1Lb, chTLR2a, chTLR2b, chTLR3, chTLR4, chTLR5, chTLR7, chTLR15, and chTLR21) have been identified. Of these, chTLR1La and chTLR1Lb, as well as chTLR2a and chTLR2b, were generated through gene duplications. chTLR2a, chTLR2b, chTLR3, chTLR4, chTLR5, and chTLR7 are mammalian TLR orthologs. Mammalian TLR8, TLR9, and TLR10 are missing in chickens, whereas TLR15 and TLR21 found in chickens do not exist in mammalian genomes. TLR15 is phylogenetically related to the TLR2 family and appears to be unique to birds.TLR21 is a non-mammalian TLR present in amphibians, fishes, and birds [8,16,17]. chTLR1La, chTLR1Lb, chTLR2a, chTLR2b, and chTLR4 recognize cell wall components from bacteria including lipoprotein, lipoteichoic acid, and lipopolysaccharide. chTLR3 recognizes double-stranded RNA from viruses. chTLR5 detects bacterial flagellin, and chTLR7 is activated by viral single-stranded RNA as well as by synthetic antiviral small molecular compounds such as imiquimod. Ligand recognition of these chTLRs is similar to their mammalian orthologs. chTLR15 recognizes virulence-associated protease [9,12,18]. Mammalian TLR9 is a cellular receptor to microbial and synthetic CpG-dideoxynucleotides-containing DNA (CpG-DNA). While TLR9 is not present in birds, chTLR21 is a functional homolog to mammalian TLR9 in terms of ligand recognition [19,20]. In addition, fish TLR21 is known to recognize CpG-DNAs [21,22,23].

Bacterial and viral DNA are potent immune stimuli. Immunostimulatory activity of these microbial DNAs is attributed to sequence motifs containing unmethylatedCpG-dideoxynucleotides in the DNA. Synthetic CpG-containing oligodeoxynucleotides (CpG-ODNs) mimic the stimulatory effect of these microbial DNA in activating immune cells [24,25,26,27]. The structure-function relationship of CpG-ODNs is better investigated in mammalian cells. In mammals, CpG-ODN activity is determined by its nucleotide length and the number of CpG-hexamer motifs, and the spacing, position, and surrounding bases of these motifs in the oligodeoxynucleotide. Moreover, studies with mammalian TLR9 revealed that the species-specific activity of CpG-ODN is largely determined by its CpG-hexamer motifs. For example, CpG-ODN with GACGTT motif displays the greatest activity toward mouse TLR9. In contrast, CpG-ODN with GTCGTT motif preferentially activates human TLR9 and TLR9 from other domestic animals including sheep, goat, horse, pig, and dog [28,29,30,31].

TLR9-mediated immunostimulatory activities of CpG-ODN have been extensively studied in mammals. CpG-ODN administration induces cytokine production, subsequently leading to maturation, differentiation, and proliferation of immune cells [25,26,32,33,34]. Because these effects facilitate both antigen-dependent and antigen-independent eradication of infected microbes, CpG-ODNs are investigated for their function as vaccine adjuvants and as antimicrobial agents [11,35,36,37,38]. Similarly, CpG-ODNs have been shown to protect chickens and ducks against bacterial and viral infections by acting as vaccine adjuvants or antimicrobial agents [12,18]. Nevertheless, TLR9 is missing in avian species, thus immunostimulatory effects observed in chicken and duck CpG-ODNs are mediated by TLR21 [8,9,16,17]. Although CpG-ODN efficacy as either a vaccine adjuvant or antimicrobial agent in birds would be determined by its immunostimulatory activity, the nucleotide sequence requirement for CpG-ODN to strongly activate avian TLR21 has not been well investigated. Here, we characterized chicken and duck TLR21s and investigated their activation by CpG-ODNs. Results showed that these two avian TLRs have a broad CpG-ODN sequences recognition profile. 

## 2. Materials and Methods

### 2.1. Approval of Animal Work 

Animal experiments were approved by the Institutional Animal Care and Use Committee (IACUC), National Health Research Institutes, Taiwan. Chicken (*Gallus domesticus*), Peking duck (*Anasplatyrhynchos* var. domestica), and white Muscovy ducks (*Cairinamoschata*) were purchased from the Animal Drugs Inspection Branch, Animal Health Research Institute (Miaoli, Taiwan), and the Livestock Research Institute (Ilan, Taiwan). These animals were handled following the guidelines.

### 2.2. Reagents and Antibodies

TRIzol reagent, SuperScript IV kit, and AccuPrime DNA Polymerases were purchased from Invitrogen (San Diego, CA, USA). RNeasy Mini Kit and QuantiNova SYBR Green PCR Kit were purchased from QIAGEN (Hilden, Germany). CpG-ODNs were purchased from Integrated DNA Technologies, Inc. Luciferase assay reagents were purchased from Promega (Madison, WI, USA). Anti-FLAG antibody and anti-actin antibody were purchased from Sigma (St. Louis, MO, USA) and Santa Cruz Biotech Inc. (Dallas, TX, USA), respectively.

### 2.3. Molecular Cloning of Chicken and Duck TLR21s cDNA

Total RNAs were purified from chicken and duck spleens using TRIzol. First-strand cDNA libraries were prepared from total RNA using SuperScriptIV first-strand synthesis kit based on the manufacturer’s instructions. To clone chicken TLR21 cDNA, forward and reverse primers (5′-atgatggagacagcggagaaggcatg-3′ and 5′-ctacatctgtttgtctccttccctgg-3′) were designed based on the coding region of chTLR21 (GenBank: JQ042914.1). cDNA containing a complete coding region of chTLR21 was cloned through PCR from the prepared chicken spleen first-strand cDNA library. To clone full-length duck TLR21 cDNA, forward and reverse primers (5′-acaggagccccccaccgccca-3′ and 5′-accccatggatggttttcctccacccca-3′) were designed based on several sequences or predicted gene sequence of avian TLR21s mRNA (GenBank: KT35043, NW_013186152.1, and NOIK01001195.1). cDNA of full-length duck TLR21 containing both 5′- and 3′- untranslated regions and a coding region was cloned through PCR from the prepared duck spleen first-strand cDNA library. The nucleotide sequence and deduced protein sequence of this duck TLR21 were submitted to GenBank (accession number MT081574).

### 2.4. Bioinformatics Analysis

Translation of nucleic acid sequences to their corresponding protein sequences was performed using NCBI Open Reading Frame Finder (https://www.ncbi.nlm.nih.gov/orffinder/). Multiple alignment of the TLR21 amino acid sequenceswas performed using Clustalw2 (http://www.ebi.ac.uk/Tools/msa/clustalw2/). Structural modeling of TLR21 ectodomains was predicted by SWISS MODEL (http://www.swissmodel.expasy.org/) using TLR9 as template.

### 2.5. Expression Vectors for Chicken, Duck, and Grouper TLR21s

Chicken and duck TLR21 expression vectors were constructed through PCR amplification of the corresponding protein-coding regions from the first-strand cDNA libraries of chicken and duck spleen. Forward and reverse primers for chicken TLR21 were 5′-atgatggagacagcggagaaggcatg-3′ and 5′-catctgtttgtctccttccctggg-3′, and primers for duck TLR21 were 5′-atggcacggccccgcccctcc-3′ and 5′-ctatgccttctcctctttctccccacgc-3′. Amplified DNA fragments were subcloned into a pEF6 vector in frame with a FLAG tag at their C-terminal ends. The expression vector for grouper TLR21 was generated as previously reported [22].

### 2.6. TLR21 Activation Assays

HEK 293 cells were grown in DMEM supplemented with 10% fetal bovine serum (FBS). Cells were co-transfected with the indicated TLR21 expression vector and a NF-κB-controlled luciferase reporter gene, treated with various CpG-ODNs as indicated, and the TLR21 activation assay was performed as previously described [22]. Relative luciferase activities were calculated as fold induction compared to unstimulated control. Data are expressed as means ± SD (*n* = 3).

### 2.7. Tissue Isolation from Chicken and Duck for First-Strand cDNA Preparation

Tissues from heart, liver, spleen, kidney, bursa of Fabricius, thymus, and lung were aseptically removed from one-week-old chickens and ducks after euthanization. Tissues were then gently minced and were soaked in TRIzol for total RNA extraction. First-strand cDNA was generated using SuperScript IV kits.

### 2.8. Preparation and Culture of Chicken and Duck Splenocytes

Spleens were aseptically removed from one-week-old chickens and ducks after euthanization. Organs were minced and pressed gently through 70 µm cell strainers. Cells were then washed and suspended in RPMI medium. The cell suspension was then carefully layered on Ficoll-Paque PREMIUM (GE Healthcare, Chicago, IL, USA), and thesplenic cell layer was separated bycentrifugation at 400× *g* for 40 min. Splenocytes were washed thrice and subsequently cultured in RPMI medium containing 10% FBS at 37°C in a humidified cell incubator with 5% CO_2_. 

### 2.9. RT-qPCR Analysis of Gene Expression

Splenocytes isolated from chicken and duck were treated with different CpG-ODNs for 4 h, and total RNA was extracted using QIAGEN RNeasy Mini Kit. Reverse transcription was performed using SuperScript IV first-strand synthesis kit. Quantitative PCR was carried out using a Roche LightCycler 480 System (Basel, Switzerland), QIAGEN, QuantiNova SYBR Green PCR Kit, and gene-specific primers. mRNA expression was normalized to GAPDH. 

### 2.10. SDS-PAGE and Immunoblot Analysis

Cells were lysed with lysis buffer containing complete protease inhibitor cocktail (Roche Life Science, Indianapolis, IN, USA). Cell lysates were resolved by SDS-PAGE and transferred to PVDF membranes. Membranes were incubated with the indicated primary antibody and then with the HRP-conjugated secondary antibody. Visualization of the immunoreactive bands was performed using chemiluminescent HRP substrate (Millipore, Temecula, CA, USA) and UVP BioSpectrum Imaging System.

### 2.11. Statistical Analysis

Data are expressed as mean ± SD. All groups were from three independent experiments. Statistical analyses were performed using Student’s *t*-test. *p* < 0.05 was considered statistically significant.

## 3. Results

### 3.1. Characterization of Chicken and Duck TLR21s

A chicken (chi) TLR21 cDNA was previously reported to encode a TLR21 protein of 972 amino acid residues [19]. Based on the expected high sequence identity between nucleotide sequences of the TLR21 gene in avian species, we designed two primers based on the 5′- and 3′-untranslated regions of goose (*Ansercygnoidesdomesticus*) and pot-billed duck (*Anaszonorhyncha*) TLR21 sequences identified from the NCBI nucleotide database to clone theduck (*Anasplatyrhynchos* var. *domestica*, Peking duck) cDNA. We therefore cloned a full-length duck (duc) TLR21 cDNA, and the sequence was submitted to GenBank (accession number: MT081574).The cDNA encodes a TLR21 protein of 976 amino acid residues, which is less than the 979 amino acid residues of the giant grouper (*Epinepheluslanceolatus*) TLR21 characterized for its interaction with CpG-ODNs [22]. 

**Figure 1:**
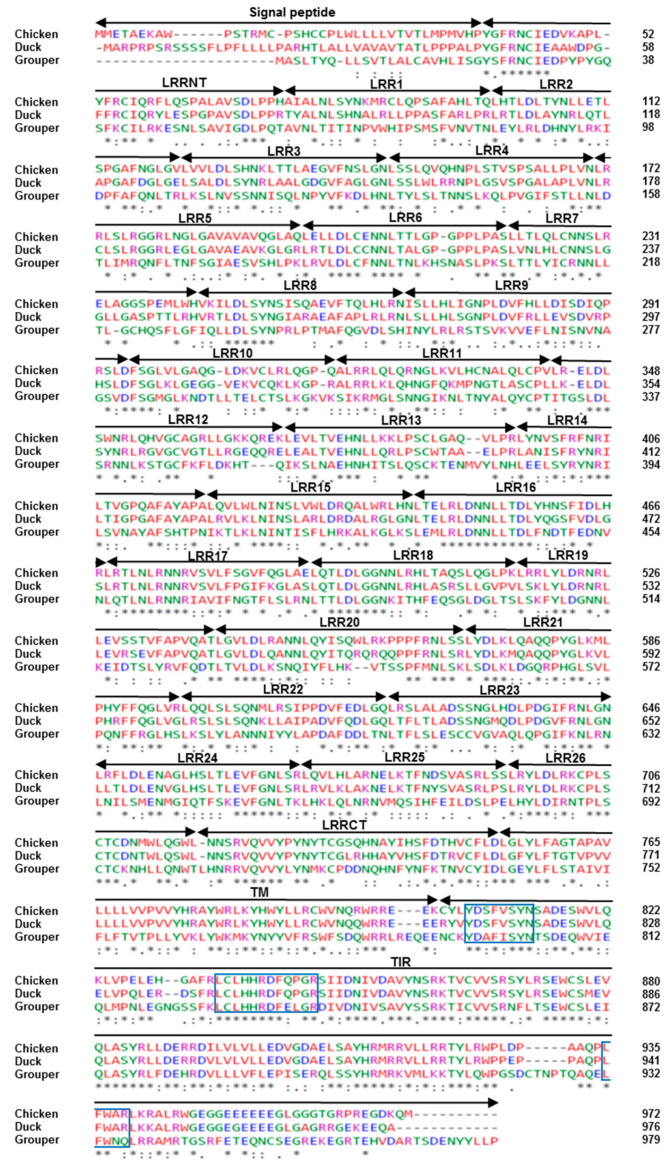
Alignment of chicken, duck, and grouper TLR21 protein sequences. Chicken and grouper TLR21 proteins were retrieved from NCBI database. The accession number is NP-001025729.1 for chicken TLR21 and AJW66342.1 for grouper TLR21. Duck TLR21 sequence was submitted to NCBI database under the accession number MT081574. Signal peptide, leucine-rich repeats (LRRs), N-terminal LRR (LRR-NT), C-terminal LRR (LRR-CT), transmembrane domain (TM), and Toll/interleukin receptor (TIR) domain are assigned based on previous reports on chicken TLR21 [39]. The boxed regions are box1, box2, and box3 in the TIR domain. Amino acids are color-coded to indicate their chemical properties: green, hydroxyl/amine/basic/Q; blue, acidic; pink, basic; red, hydrophobic (including aliphatic Y). Asterisk, identical residues; two dots, highly conservative substitutions; single dot, conservative substitutions.

**Figure 2:**
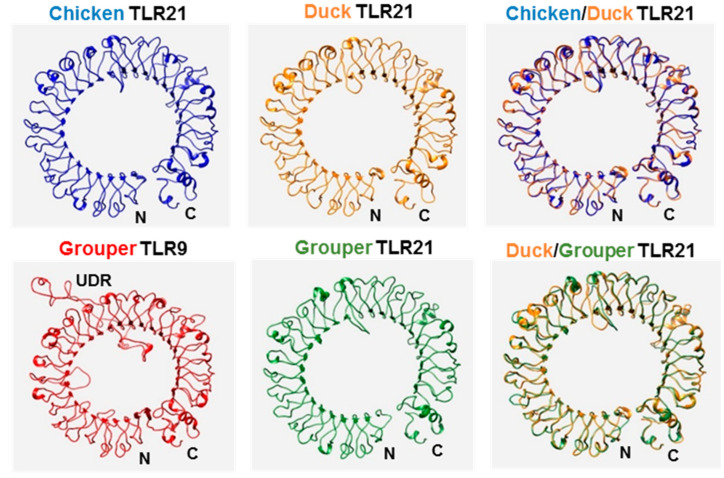
Computational modeling of the ectodomain protein structures of TLR21 from chicken, duck, and grouper. The ectodomain protein structures of TLR21 and TLR9 from different species and their superimpositions, as indicated, were predicted with SWISS MODEL (http://www.swissmodel.expasy.org/). N: N-terminal end, C: C-terminal end of the ectodomain. UDR: undefined region.

These three TLR21s contain an extracellular domain, a transmembrane domain, and a Toll/IL-1 (TIR) cytosolic domain, and they have N-terminal leucine-rich repeat (LRR-NT), leucine-rich repeats (LRRs) and a C-terminal leucine-rich repeat (LRR-CT) in their ectodomain. The TIR domain is better conserved among these TLR21s. In addition, the three boxes in the TIR domain for mammalian TLR signaling are conserved in TIR domains of these TLR21s (Figure 1). Ligand binding occurs at TLR ectodomains, therefore, we used SWISS MODEL software to compare predicted three-dimensional ectodomain structures of chicken, duck, and grouper TLR21s. Ectodomains of these TLR21s have relatively similar horseshoe-shaped solenoid three-dimensional structures. A previous study revealed that fish TLR9s contain an undefined region (also called as Z-loop) in their ectodomains, whereas their functional homolog, TLR21s, do not [23]. Analysis of chicken and duck TLR21s did not show an undefined region in their ectodomain (Figure 2), indicating that this fish TLR21 feature is preserved in these two avian TLR21s. 

### 3.2. Phylogenetic Analysis of Chicken and Duck TLR21s

**Figure 3:**
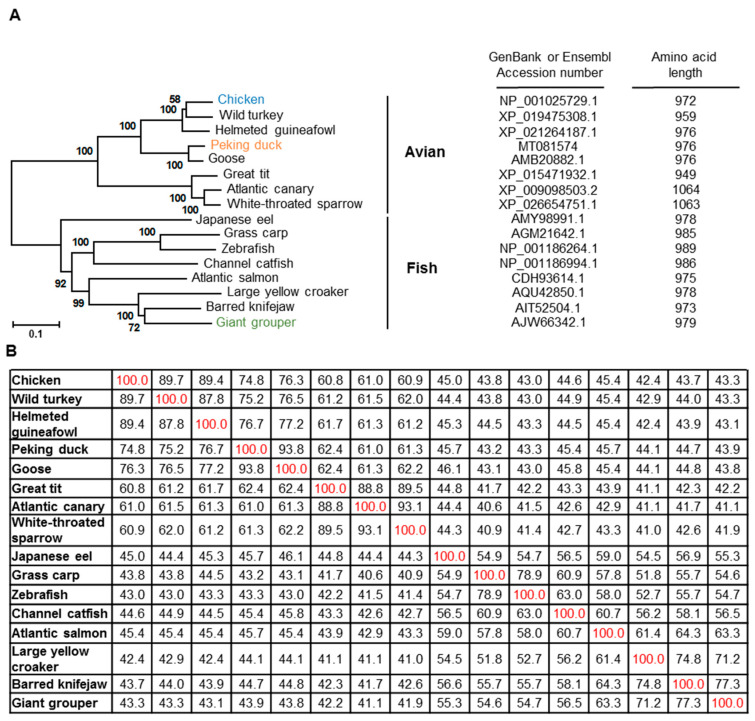
Phylogenetic and protein identity analyses of avian and fish TLR21s. (**A**) Phylogenetic analysis. Left panel shows the phylogenetic tree of these TLR21s. Right panel shows GenBank accession numbers of these TLR protein sequences (left column). Numbers in the right column are the amino acid lengths of these TLRs. (**B**) Protein identity analysis. Numbers in the table represent percentage protein identities of each TLR (red) to each other (black). In the database, the hypothetical protein sequences of wild turkey, helmeted guinea fowl, great tit, Atlantic canary, and white-throated sparrow are annotated as TLR13, though, avian species do not contain TLR13. It is likely that these hypothetical proteins are TLR21 but were mistakenly annotated as TLR13 [40].

Vertebrate TLRs are divided into 6 families, namely, family 1, 3, 4, 5, 7, and 11. The TLR11 family contains two subfamilies, TLRs 11–13 and TLRs 20–22. TLR21 is an ortholog of mouse TLR13 [39,40]. When searching NCBI nucleotide databases, putative sequences for the TLR13 of avian species including wild turkey, helmeted guinea fowl, great tit, Atlantic canary, and white-throated sparrow were identified, but none of their TLR21s were found. As birds are not reported to have TLR13, whether these putative sequences were TLR21s that were mistakenly annotated as TLR13s requires clarification [40]. Phylogenetic and protein identity analyses using the protein sequences of these TLR21s and hypothetical TLR13s from different avian and fish species using ClustalW2 revealed that the ducTLR21 protein sequence is closely related to protein sequences in avian species, having 93.8%, 74.8%, and 61.3% protein identity to the goose, chicken, and sparrow sequences. ducTLR21 has 43.9% protein identity to grouper TLR21 and has around 43–46% protein identities to various fish TLR21 sequences. Generally, TIR domains are better conserved among different TLRs. Consistently the protein identity of chiTLR21 TIR domain to the TIR domains of duck and grouper are 86.3% and 56.6%, respectively (Figure 3).

### 3.3. Tissue Distribution of TLR21 in Chicken and Duck and Activation Their Splenocytes by CpG-ODNs

**Figure 4:**
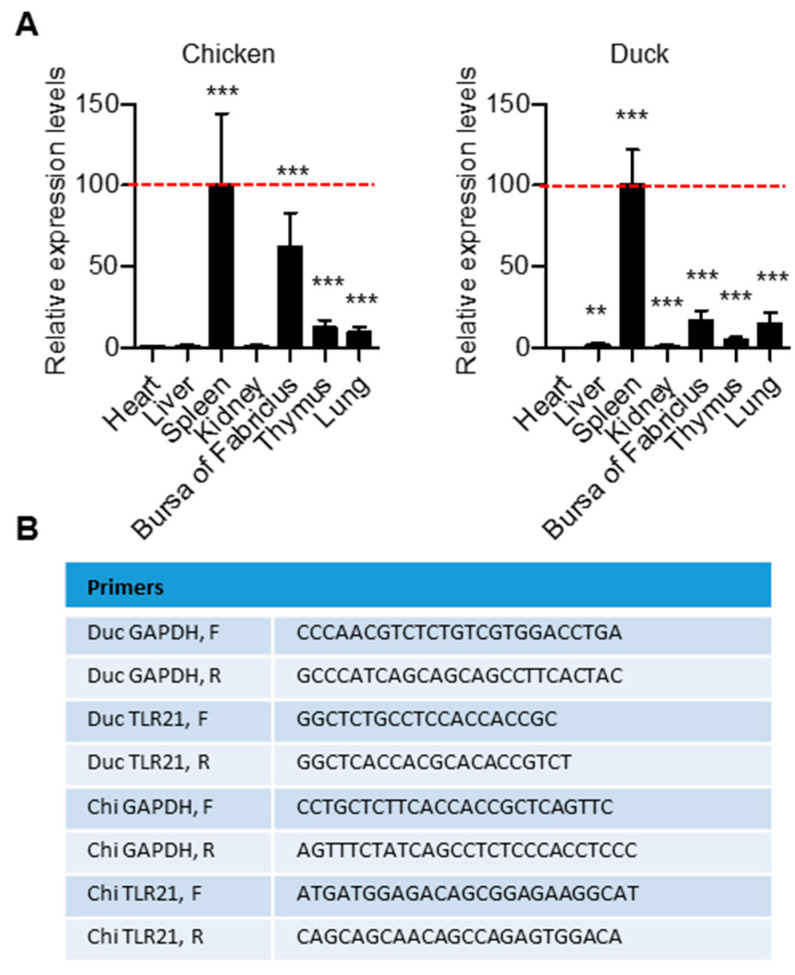
Expression of TLR21 in different tissues of chicken and duck.Tissue distributions of TLR21 in chicken and duck were analyzed through RT-qPCR. (**A**) Expression levels of TLR21 in different tissues were compared with the levels in spleen (dotted red line). Data show relative TLR21 expression levels to the expression in spleen, and represent means ± SD (*n* = 3). ** *p* < 0.01, *** *p* < 0.001 compared with control. (**B**) Sequences of primers used in this study for PCR amplification of specific genes.

**Figure 5:**
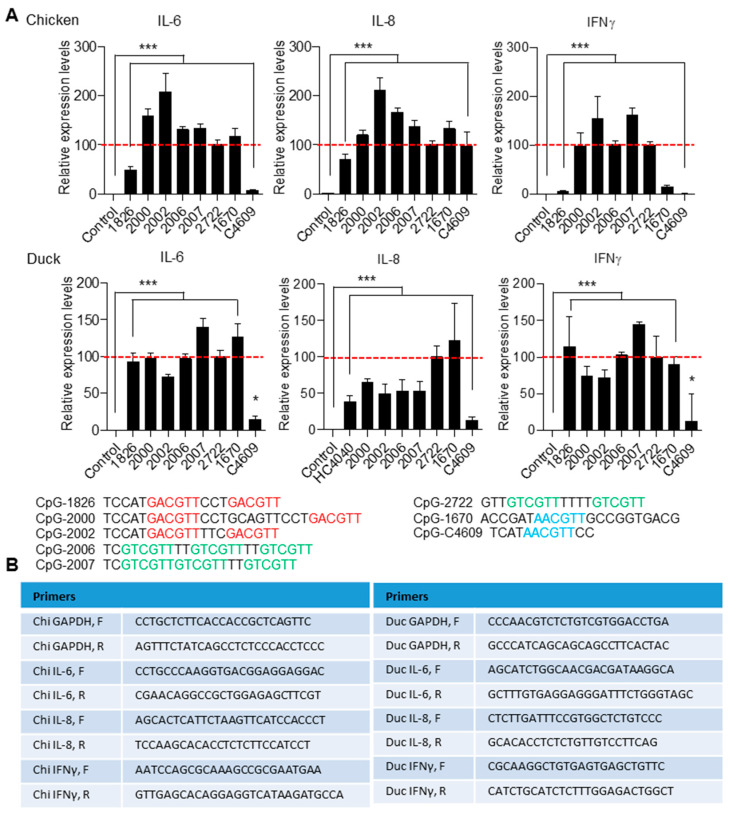
Induction of cytokine expression in chicken and duck splenocytes by different CpG-ODNs. (**A**) Expression of IL-6, IL-8, and IFNγ in chicken and duck splenocytes treated with 0.8 μM of different CpG-ODNs as indicated for 4 h and analyzed with RT-qPCR. Data show related cytokine expression levels to the expression induced by CpG-2722 (dotted red line), and represent means ± SD (*n* = 3 independent experiments). * *p* < 0.05, *** *p* < 0.001 compared with control. Sequences of CpG-ODNs are shown below the figures. (**B**) Sequences of primers used in this study for PCR amplification of specific genes.

TLR21 expression in heart, liver, spleen, kidney, bursa of Fabricius, thymus, and lung tissues of chickens and ducks were analyzed by RT-qPCR. chiTLR21 had higher expression in the spleen and bursa of Fabricius, and modest expression in the thymus and lung. ducTLR21 had strongest expression in the spleen and modest expression in the bursa of Fabricius, thymus, and lung, and was weakly expressed in the kidney and liver. These revealed that chicken and duck TLR21s are expressed in immune-relevant tissues (Figure 4). We further investigated induction of cytokine production in chicken and duck cells by different CpG-ODNs. For this, splenocytes were purified from chickens and ducks and treated with CpG-ODNs with different sequences and different CpG-hexamer motif types including GACGTT, GTCGTT, and AACGTT; induction of IL-6, IL-8, and IFNγ was then analyzed by RT-qPCR. Results revealed that these CpG-ODNs have different activities to the chicken and duck splenocytes. Nevertheless, regardless of which CpG-hexamer motif type they possess, these CpG-ODNs were able to activate cytokine production in chicken and duck splenocytes(Figure 5). This activation profile by CpG-ODNs is quite different from that of some fish TLR21s, previously shown to preferentially respond to CpG-ODNs with a GTCGTT hexamer motif [21,22,23].

### 3.4. Broad CpG-ODN Sequence Recognition of Chicken and Duck TLR21s

**Figure 6:**
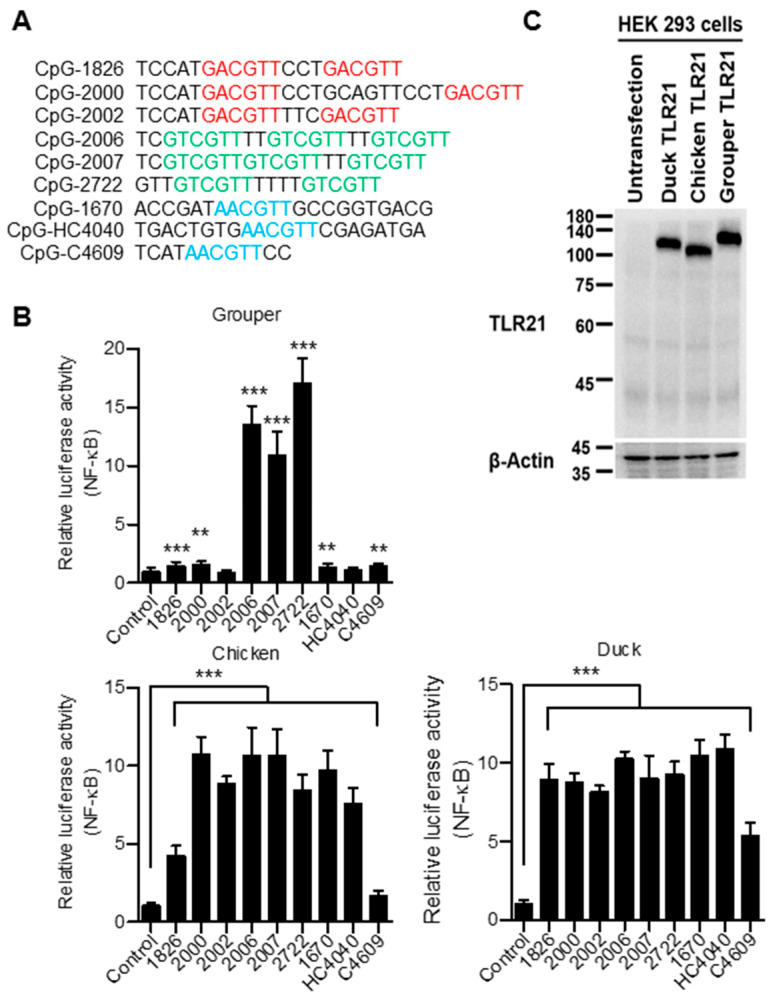
Activation of chicken, duck, and grouper TLR21s by different CpG-ODNs. Human embryonic kidney (HEK) 293 cells were co-transfected with a control vector or expression vector for different TLR21s as indicated, along with a nuclear factor (NF)-κB controlled luciferase reporter gene, and treated with 0.8 μM of CpG-ODN for 7 h. (**A**)Sequences of CpG-ODNs used in this study. Different types of CpG-hexamer motif are shown with different color. (**B**) Relative luciferase activities of the treated cells. Data represent means ± SD (*n* = 3 independent experiments). ** *p* < 0.01, *** *p* < 0.001 compared with the control. (**C**) Immunoblot analysis of the TLR21 expression in HEK293 cells. β-actin was blotted as a loading control.

Because the splenocytes are composed by different cell types that is not favorable for a precise study of the CpG-ODNs activities, we then use a cell-based TLR21 activation assay to investigate activation of chicken and duck TLR21s by different CpG-ODNs and compared their activation profiles with that of grouper TLR21. For this, the cell-based TLR21 activation assay was established by co-transfecting an expression vector for chicken, duck, or grouper TLR21 and a NF-κB-driven luciferase reporter gene into HEK293 cells. These were stimulated with CpG-ODNs with different sequence and different CpG-hexamer motif types. Distinct from the preferential activation of grouper TLR21 by CpG-ODNs with the GTCGTT hexamer motif, chicken and duck TLR21s were activated by CpG-ODNs with different types of hexamer motifs (Figure 6). This is consistent with the abilities of these CpG-ODNs to activate chicken and duck splenocytes (Figure 5), suggesting that chicken and duck TLR21s have broad CpG-ODN sequence recognition profiles.

### 3.5. Chicken and Duck TLR21s Do Not Distinguish Different Types of CpG-hexamer Motifs

**Figure 7:**
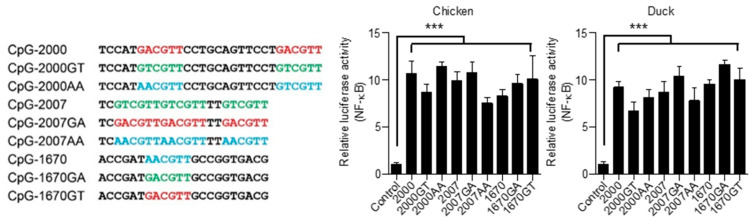
Activation of chicken and duck TLR21s by CpG-ODNs with exchanged CpG-hexamer motifs. Human embryonic kidney (HEK) 293 cells were co-transfected with a control vector or expression vector for different TLR21s as indicated, along with a nuclear factor (NF)-κB controlled luciferase reporter gene, and treated with 0.8 μM of CpG-ODN for 7 h. Luciferase activities in the treated cells were measured. Data represent means ± SD (*n* = 3 independent experiments). *** *p* < 0.001 compared with control. Sequences of CpG-ODNs used in this study are shown on the left.

The CpG-hexamer motif type is essential in determining species-specific activity of CpG-ODN to mammalian TLR9s. Human TLR9 responds to CpG-ODNs with the GTCGTT motif, whereas mouse TLR9 is strongly activated by CpG-ODNs with GACGTT or AACGTT, but is only weakly activated by CpG-ODN with GTCGTT [28,29,30]. Furthermore, previous studies and Figure 6C show that zebrafish and grouper TLR21s preferentially responded to CpG-ODNs with a GTCGTT motif [21,22]. To confirm that chicken and duck TLR21s have a broad recognition profile to different type of CpG-hexamer motifs, CpG-ODNs with the same nucleotide sequence and length but with different type of CpG-hexamer motifs were designed for TLR21 activation. These CpG-ODNs, including CpG-2000 containing GACGTT motifs, CpG-2722 containing CTCGTT motifs, CpG-1670 containing AACGTT motifs, and their derivatives, generated by replacing their CpG-hexamer motif with different type of CpG-hexamer motifs, are shown in Figure 7. When treating chicken and duck TLR21-expressing cells with these CpG-ODNs, these CpG-ODNs did not display significant differences in their chiTLR21 and ducTLR21 activation capability (Figure 7). These results reveal that chicken and duck TLR21s do not distinguish different types of CpG-hexamer motifs as mammalian TLR9s and grouper TLR21 do. 

### 3.6. Responsiveness of Chicken and Duck TLR21s to CpG-ODNs with Different Lengths and Varied Spacing between Their CpG-hexamer Motifs

**Figure 8:**
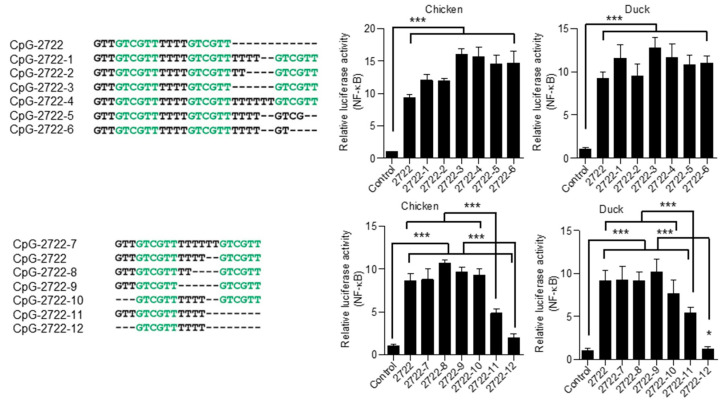
Activation of chicken and duck TLR21s by CpG-ODNs with different length and spacing between CpG-hexamer motifs. Human embryonic kidney (HEK) 293 cells were co-transfected with a control vector or expression vector for different TLR21s as indicated, along with a nuclear factor (NF)-κB controlled luciferase reporter gene. Cells were treated with 0. 8 μM of CpG-ODN for 7 h. Luciferase activities in the cells were measured. Data represent means ± SD (*n* = 3 independent experiments). * *p* < 0.05, *** *p* < 0.001 compared with the control. Sequences of CpG-ODNs used in this study are shown on the left.

Previous studies revealed that CpG-ODN length and the spacing between two CpG-hexamer motifs of a CpG-ODN can determine CpG-ODN activity. For example, CpG-C4609 with 12 nucleotides more strongly activates rabbit TLR9 than CpG-2007 and CpG-1826, which contain 22 and 20 nucleotides, respectively [41]. CpG-2722, with four thymidines between two GTCGTT motifs, has stronger activation of grouper TLR21 than CpG-272 and CpG-2721, which contain two thymidines and no spacing between the two GTCGTT motifs, respectively [22]. Therefore, we further investigated the responsiveness of chicken and duck TLR21s to CpG-ODNs with different lengths and varied spacing between CpG-hexamer motifs. CpG-2722 contains 19 nucleotides; based on this, different CpG-ODNs lengthened by adding a GTCGTT motif to the 3′-end and adding thymidine spacing between the second and third GTCGTT motifs were designed. Chicken and duck TLR21-expressing cells were treated with these CpG-ODNs. Results showed no major change in the stimulatory activity of these CpG-ODNs with nucleotide length increased from 19 to 31 (Figure 8). Also, thymidine spacing between the first and second GTCGTT motifs were adjusted, and 5′ or 3′ nucleotides were trimmed to generate CpG-ODNs shorter than CpG-2722, and their activities were analyzed. Results showed that compared to CpG-2722 activity, no major change was observed for the activities of CpG-2722-7, -8, -9, and -10, which have 21, 17, 15, and 16 nucleotides, respectively. Nevertheless, activities of CpG-2722-11 and -12, comprising only 10 and 13 nucleotides, were reduced (Figure 8). Overall data suggested that chicken and duck TLR21s are activated by CpG-ODNs with lengths from 15 to 31 nucleotides, and spacing between the two GTCGTT motifs does not play a role in determining activity.

### 3.7. Requirement of CpG-dideoxynucleotides for Activation of Chicken and Duck TLR21s

**Figure 9:**
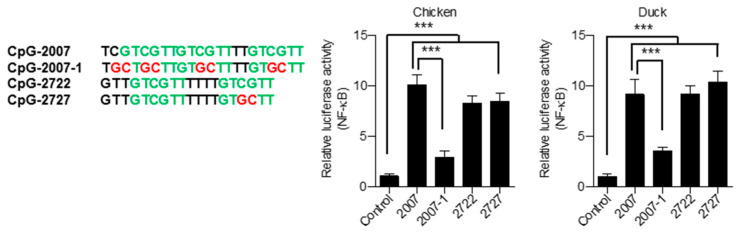
Activation of chicken and duck TLR21s by CpG-ODNs with reversed CpG-didexoxynucleotides. Human embryonic kidney (HEK) 293 cells were co-transfected with a control vector or expression vector for different TLR21s as indicated, along with a nuclear factor (NF)-κB-controlled luciferase reporter gene. Cells were treated with 0.8 μM of CpG-ODN for 7 h. Luciferase activities in the cells were measured. Data represent means ± SD (*n* = 3 independent experiments). *** *p* < 0.001 compared with control. Sequences of CpG-ODNs used in this study are shown on the left.

CpG-dideoxynucleotides in a CpG-hexamer motif are required for CpG-ODN activation of mammalian TLR9s [28,29,30]. CpG-2722 contains two copies of CpG-hexamer motifs, and previous studies revealed that the second copy of the CpG-hexamer motif at the 3′ end of CpG-2722 is not required for activating grouper TLR21, since CpG-2727, containing a reversed CpG-dideoxynucleotide in its second copy of the CpG-hexamer motif, had similar activity in grouper TLR21 activation as CpG-2722 [22]. To investigate this property of CpG-dideoxynucleotides and the number of CpG-hexamer motifs required for CpG-ODN to activate chicken and duck TLR21s, CpG-2007-1 was generated in which all CpG-dideoxynucleotides in CpG-2007 were reversed. Chicken and duck TLR21s were stimulated with CpG-2007, -2007-1, -2722 and -2727. Results revealed that CpG-2727 had activity as strong as CpG-2722 in activating chicken and duck TLR21s, whereas CpG-2007-1 activity was reduced (Figure 9), indicating that at least one copy of the CpG-hexamer motif with CpG-dideoxynucleotides is required for CpG-ODN to strongly activate chicken and duck TLR21s.

## 4. Discussion

CpG-ODNs are potent immunostimulants investigated as anti-infectious agents and vaccine adjuvants for various species [11,35,36,37,38]. In mammals, their cellular receptor is TLR9, in avian species, TLR21, and fishes have both TLR9 and TLR21 [19,20,21]. CpG-ODN immunostimulatory activity is determined by structures including its CpG-hexamer motif type, spacing between motifs, nucleotide sequence, and length [28,29,30,31]. While the structural-functional relationship for the interaction between CpG-ODNs and TLR9 has been investigated, interaction between CpG-ODNs and TLR21 is not well-known. Moreover, it is unclear whether there are differences between the functional properties of avian and fish TLR21s. In this study, we characterized chicken and duck TLR21s and investigated the structural requirements for CpG-ODN to strongly activate these two poultry TLRs.

Computer modeling revealed that chicken and duck TLR21s have a horseshoe-like ectodomain similar to other TLRs. Also, similar to fish TLR21s, these two do not contain an undefined region similar to that in between LRR14 and LRR15 of its functional homolog, TLR9. Of the mammalian TLRs, TLR7, and TLR8 are most closely related to TLR9, and these three all contain an undefined region in their ectodomain [42,43,44]. Three rodent TLR8s, namely, mouse, rat, and rabbit TLR8s, are relatively insensitive to ligand stimulations, and a varied undefined region in the ectodomain of these three TLRs was suggested to cause the low responsiveness of these TLR8s to ligand stimulation [45,46]. In fishes, varied undefined regions were found in TLR9s, but TLR21s do not contain this undefined region, leading to speculation on whether TLR21 is the major receptor for CpG-DNA in most fishes and whether TLR9 may have low activity or even be nonfunctional in some fishes [23]. TLR9 is not present in avian species. Of the ten avian TLRs, TLR21 is the only TLR that recognizes CpG-DNA. Thus, there is significance for TLR21 to be selected through evolution to ensure detection of pathogens with microbial DNA by avian species.

Various studies showed that mammalian TLR9s have preferences in terms of recognizing different CpG-ODN nucleotide sequences. Optimal CpG-hexamer motifs for activating rodent TLR9, including mouse and rabbit, are GACGTT and AACGTT, and for activating TLR9 in humans and other domestic animals, the optimal motif is GTCGTT [28,29,30,31]. Similarly, CpG-ODNs with a GTCGTT motif have stronger activities in activating zebrafish and grouper TLR21s than CpG-ODNs with GACGTT motif [21,22]. In addition, nucleotide length also plays a role in determining CpG-ODN stimulatory activity. Generally, 18–24 nucleotides are required for a CpG-ODN to strongly activate mouse and human TLR9s. Nevertheless, CpG-ODNs that are 12–14 nucleotides long show stronger activities to rabbit TLR9 [41]. Our results show that chicken and duck TLR21s are strongly activated by CpG-ODNs with GACGTT or GTCGTT motif, indicating that these TLRs do not distinguish different types of CpG-hexamer motifs. Furthermore, chicken and duck TLR21s are activated by CpG-ODNs with different lengths (15–31 nucleotides) and spacing between their CpG-hexamer motifs.Thus, compared to mammalian TLR9s and fish TLR21s, chicken and duck TLR21s have broad ligand recognition profiles to different CpG-ODN sequences and lengths. 

Major infectious diseases of poultry birds including Salmonellosis, Coccidiosis, *Campylobacter* infections, avian influenza, infectious bronchitis, Marek’s disease, infectious bursal disease, and Newcastle disease, can cause large economic losses in the industry. Some bird-borne microbes can spread to humans and threaten human health. Therefore, there is a need to prevent poultry infectious diseases [1,2,3,47,48]. Vaccination is commonly used to protect humans and other species against microbial infections. While vaccines have been used in poultry farming to reduce infectious diseases, various disadvantages exist for some conventional vaccines and adjuvants including virulence reversion of live attenuated or inactivated vaccines, and the toxicity and poor ability of traditional adjuvants to induce optimal immune responses. Adjuvants such as Freund’s adjuvant frequently induce strong side effects resulting in abscesses and granulomas at the injection site. Although aluminum salt significantly enhances serum humoral response when supplemented to vaccines, the capability of this adjuvant to induce cell-mediated immune response is poor [49,50,51,52]. 

CpG-1018 has been used as adjuvant in a Hepatitis B vaccine approved by the US FDA in 2017. This vaccine was proven to be more effective than aluminum salt-adjuvanted Hepatitis B vaccines [53,54], suggesting that CpG-ODN is a potent and safe adjuvant. When formulated with antigens, CpG-ODNs can increase survival of poultry birds challenged with various viruses and bacteria for microbial infectious diseases, including avian influenza, Newcastle disease virus, infectious bursal disease virus, *Salmonella*, and *E. coli*, by increasing cytokine production, lymphocyte proliferation, and serum IgG. While the CpG-ODNs with AACGTT motif have not yet well investigated, these CpG-ODNs shown to have adjuvant activity contain either CTCGTT or GACGTT motifs. Furthermore, in chickens, CpG-ODN can be administered through different routes including oral, intranasal, subcutaneous, and in ovo injections [55,56,57,58,59,60,61,62,63,64,65,66]. Thus, CpG-ODN development has increased the strategies for designing adjuvanted vaccines for poultry birds. Our studies show that chicken and duck TLR21s have a broad CpG-ODN sequence recognition profile, revealing that there are more choices of CpG-ODNs for optimal use as adjuvants in vaccines to boost antigen-dependent immune responses in poultry birds.

## 5. Conclusions

TLR21 is a pattern recognition receptors for detection of microbial DNA to initiate host response to infections. In addition, it is the cellular receptor to mediate the anti-infectious and adjuvant activities of CpG-ODNs in avian. The usage of CpG-ODN as vaccine adjuvant for poultry birds is been investigated [10,11,12,16,17]. Our results in this study are a novelty in describing the CpG-ODN recognition feature of chicken and duck TLR21s. Unlike the mammalian TLR9s and some fish TLR21s, these two avian TLR21s recognize a broad array of CpG-ODN sequences for their activation. This suggests that the innate immune system of chicken and duck have a strong ability to sense DNA-associated molecular patterns from microbes to initiate immune responses for host defense to infections. In addition, the result also suggests that there are more CpG-ODN choices for using as immune stimulatory agent (such as vaccine adjuvant) for poultry bird than available for other species.
